# Embracing Large Language Models for Adult Life Support Learning

**DOI:** 10.7759/cureus.75961

**Published:** 2024-12-18

**Authors:** Serena Patel, Rohit Patel

**Affiliations:** 1 General Surgery, Imperial College NHS Trust, Ilford, GBR; 2 Oral and Maxillofacial Surgery, Kings College Hospital, London, GBR

**Keywords:** advanced life support, bard, basic life support bls, chatgpt, google bard, large language models (llm), resus

## Abstract

Background

It is recognised that large language models (LLMs) may aid medical education by supporting the understanding of explanations behind answers to multiple choice questions. This study aimed to evaluate the efficacy of LLM chatbots ChatGPT and Bard in answering an Intermediate Life Support pre-course multiple choice question (MCQs) test developed by the Resuscitation Council UK focused on managing deteriorating patients and identifying causes and treating cardiac arrest. We assessed the accuracy of responses and quality of explanations to evaluate the utility of the chatbots.

Methods

The performance of the AI chatbots ChatGPT-3.5 and Bard were assessed on their ability to choose the correct answer and provide clear comprehensive explanations in answering MCQs developed by the Resuscitation Council UK for their Intermediate Life Support Course. Ten MCQs were tested with a total score of 40, with one point scored for each accurate response to each statement a-d. In a separate scoring, questions were scored out of 1 if all sub-statements a-d were correct, to give a total score out of 10 for the test. The explanations provided by the AI chatbots were evaluated by three qualified physicians as per a rating scale from 0-3 for each overall question and median rater scores calculated and compared. The Fleiss multi-rater kappa (κ) was used to determine the score agreement among the three raters.

Results

When scoring each overall question to give a total score out of 10, Bard outperformed ChatGPT although the difference was not significant (p=0.37). Furthermore, there was no statistically significant difference in the performance of ChatGPT compared to Bard when scoring each sub-question separately to give a total score out of 40 (p=0.26). The qualities of explanations were similar for both LLMs. Importantly, despite answering certain questions incorrectly, both AI chatbots provided some useful correct information in their explanations of the answers to these questions. The Fleiss multi-rater kappa was 0.899 (p<0.001) for ChatGPT and 0.801 (p<0.001) for Bard.

Conclusions

The performances of both Bard and ChatGPT were similar in answering the MCQs with similar scores achieved. Notably, despite having access to data across the web, neither of the LLMs answered all questions accurately. This suggests that there is still learning required of AI models in medical education.

## Introduction

The advent of artificial intelligence (AI) since the 20th century has resulted in the development of machines with the capabilities of executing human intelligence processes to enable problem-solving and simplification of tasks. Since their conception, machine learning algorithms and neural networks have become more advanced with technologies transforming the way we work in industries ranging from manufacturing to medicines to finance [1]. Within medicine, the evolution of neural networks and deep learning has led to automated clinical image recognition and interpretation, offering the potential to transform radiomics and aid histopathological diagnosis [2,3] with high sensitivity and high specificity [4]. In the context of clinical education, AI has evolved to enable virtual patient systems [5], virtual-reality and robotic surgical simulation applications [6], and to support distance learning. Indeed, with medicine becoming increasingly digitalised, harnessing the power of AI to augment learning is crucial to ensure that students acquire the digital literacy needed to incorporate technology into their future practice and effectively utilize the data resources available to them [7].

Both ChatGPT and Bard are large language model (LLM) generative AI chatbots trained on large datasets to recognize, predict, translate, or generate text. As such, they can be trained to problem-solve through document summarisation, text classification, question answering, and text generation. Their use in clinical education has been suggested in medical answering and reasoning, assignment feedback, simulated patient chatbots, and learning clinical reasoning [8]. ChatGPT, also known as Chat Generative Pre-trained Transformer, developed by OpenAI and originally released to the public in November 2022 (based on data pre-September 2021), has different versions trained on different datasets. It has already been shown to have a role in supporting healthcare professionals with medical education and medical writing [9]. Bard is a newer chatbot, developed and released by Google in March 2023 [10], and has real-time access to data through Google’s search engine. Similar to ChatGPT, Bard has the ability to generate text with conversational responses with a potential to impact medical education.

The Resuscitation Council UK [11] is a national charitable organisation responsible for setting standards for cardiopulmonary resuscitation (CPR) and related disciplines in the UK. In addition, the organisation is responsible for providing mandatory life support training courses including intermediate and advanced life support for healthcare professionals including doctors, dentists, nurses, and midwives for the recognition and management of deteriorating patients and appropriate response to cardiac arrest events. Completion of life support qualifications is mandatory for clinical medical students and junior doctors and courses involve pre-reading as well as an in-person or e-learning course that delegates must do to achieve certification. Several of these clinical courses provide pre-course test questions to aid student learning and post-course tests that delegates must pass for certification.

Previous studies have assessed the utility of LLMs in answering clinical medical questions in various disciplines (pharmacology, microbiology, pathology, clinical knowledge) in medical licensing examinations [12-14]. This study aimed to evaluate the efficacy of LLM ChatGPT and Bard in answering resuscitation-based medical questions, relating to causes and management of cardiac arrest. We assessed the accuracy of responses and the quality of explanations to evaluate the utility of LLMs as educational tools to support healthcare professionals in their preparation for life support courses and their understanding of the assessment and management of unwell patients, including response in cardiac arrest.

## Materials and methods

Artificial Intelligence

ChatGPT-3.5 (Open AI, San Fransisco, USA) and Bard (Google, Mountain View, USA), both freely accessible to the public, were the AI chatbots utilised in this study. ChatGPT used the Generative Pre-Trained Transformer 3.5 whilst Bard utilised the Language Model for Dialogue Applications (LaMBDA). At the time of this study, ChatGPT-3.5 was trained and confined to datasets up to 2021, limiting access to the latest research. In contrast, Bard had the capability of searching the Internet in real-time to find the most recent research on a topic. Both AI chatbots allow text input and provide text output to answer questions and provide explanations.

Test questions

The performance of the AI chatbots was tested on true-false test questions taken from an Intermediate Life Support pre-course question test developed by the Resuscitation Council UK. Thus, all inputs represent true training questions for cardiopulmonary resuscitation teaching in the United Kingdom and are essential knowledge for the performance of cardiopulmonary resuscitation by healthcare professionals in the United Kingdom. The main questions had a similar structure each consisting of a stem with four true-false sub-questions (a-d), with the chatbots asked to choose all correct options and then confirm. These constituted 10 main questions, which with the sub-questions totalled 40 true-false questions.

Testing

All inputs into LLMs occurred on 4th October 2023 by a single tester with the other author observing. Both ChatGPT and Bard were given the instruction to ‘act as a medical doctor’ to answer the questions. The questions were submitted by reproducing the original Resuscitation Council UK’s question verbatim. For each question, the chatbot was advised to ‘Choose ALL correct options and then confirm’ as in the original question. The answers selected by the chatbots were compared to the true answers to the questions on the Resuscitation Council UK’s pre-course test paper. Scoring occurred in two ways. The 40 sub-questions were tested to give a total score out of 40, with 1 point scored for each accurate response to each statement a-d. In a separate scoring, each of the 10 main questions was scored 1 if all the responses to sub-questions a-d were correct, to give a total score out of 10.

The explanations provided by the AI chatbots were evaluated by three qualified physicians as per a rating scale from 0-3 for each overall question. All physicians have medical degrees from the United Kingdom, hold advanced life support certification from the Resuscitation Council UK, and have experience teaching undergraduate medicine. A score of zero denoted the explanation was wholly incorrect, providing no insight or knowledge; a score of one was used when the explanation had some correct or useful information but lacked significant detail or contained errors; a score of two was used when the explanation was correct and relevant but lacked detail or did not provide a sufficient nuanced explanation for all options; a score of three was given for clear, comprehensive, and correct explanations which sufficiently explained all of the options for each question. The median score for each question was calculated and rounded to the nearest integer for analysis.

Ethical approval

Ethical approval was not required for this study as there were no animal or human subjects. 

Data analysis

Data was inputted into Microsoft Excel 365 (Microsoft Corporation, Redmond, USA). Statistical analysis was conducted using R (version 4.3.1, R Foundation for Statistical Computing, Vienna, Austria). The Chi-squared non-parametric test was used to determine the association of correct answers between ChatGPT and Bard. The Fleiss kappa test was used to assess score agreement between the three raters. Results were statistically considered statistically significant if p < 0.05. 

## Results

Accuracy of answers

**Figure 1:**
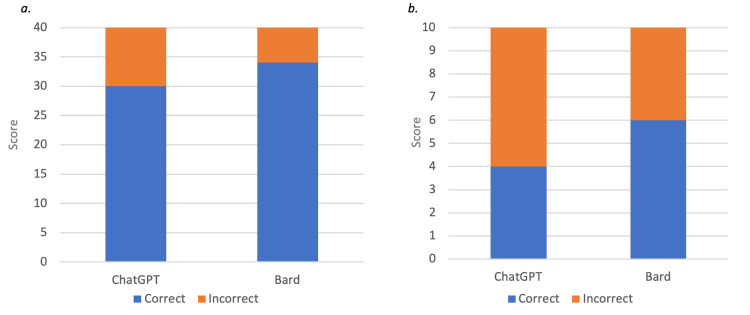
Comparison of performance of ChatGPT and Bard when scoring a) out of 40 b) out of 10.

In assessing the accuracy of correct answers to the questions, the performances of both Bard and ChatGPT were noted to be similar. When scoring the main questions to give a total score out of 10, Bard outperformed ChatGPT although the difference was not significant (6 vs 4, p=0.37). Furthermore, there was no statistically significant difference in the performance of ChatGPT compared to Bard when scoring each sub-question separately to give a total score out of 40 (30 vs 34, p=0.26). The total scores achieved by both ChatGPT and Bard on the tests are shown in Figure 1.

**Figure 2:**
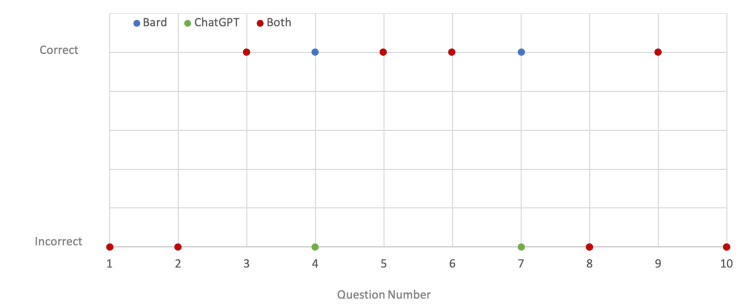
Correct and incorrect responses by question number

Interestingly, of the 10 main questions, all questions answered incorrectly by Bard were also answered incorrectly by ChatGPT, with additional questions also answered incorrectly by ChatGPT. Four questions were answered correctly by both LLMs, four questions were answered incorrectly by both LLMs, and two questions were answered correctly by Bard and incorrectly by ChatGPT. Figure 2 shows the correct and incorrect responses by main question number for each of the LLMs tested.

**Figure 3:**
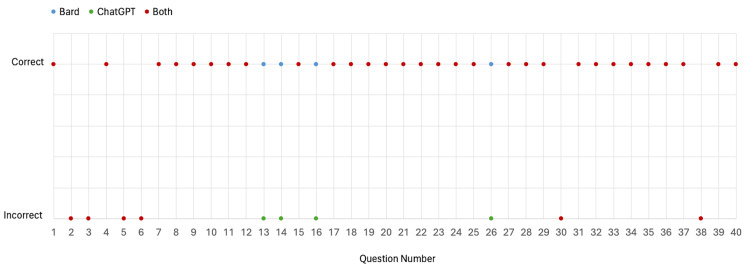
Correct and incorrect responses for each of the 40 sub-questions

When considering the 40 sub-questions, a similar pattern was noted. All questions answered incorrectly by Bard were also answered incorrectly by ChatGPT, and additional questions that were correct by Bard were incorrect by ChatGPT. Thirty questions were answered correctly by both LLMs, six were answered incorrectly by both LLMs, and four were answered correctly by Bard but incorrectly by ChatGPT. This is shown in Figure 3.

Quality of explanations

**Figure 4:**
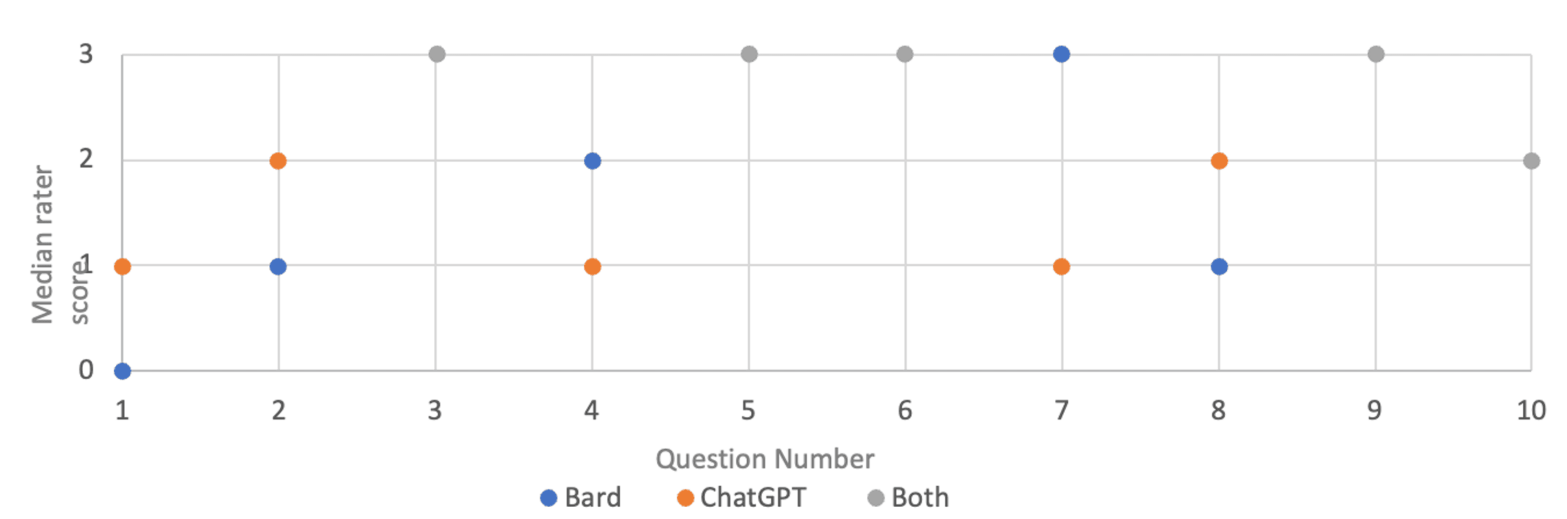
Median rater scores for each chatbot for each main question

Explanations provided by ChatGPT and Bard for each main question were scored out of 3. The median rater scores for each question are shown in Figure 4. Both ChatGPT and Bard had the same median rater score for five questions, four of these had a median rater score of 3, and one had a median rater score of 2. In three cases, the median rater score was higher for ChatGPT than Bard and in two cases, the score was higher for Bard than ChatGPT.

Important to note is that despite answering certain questions incorrectly, both AI chatbots provided some useful correct information in their explanations of the answers to these questions. The Fleiss multi-rater kappa was 0.899 (p<0.001) for ChatGPT and 0.801 (p<0.001) for Bard. This shows strong inter-rater reliability for both AI chatbots in providing explanations for the answers to the questions.

**Figure 5:**
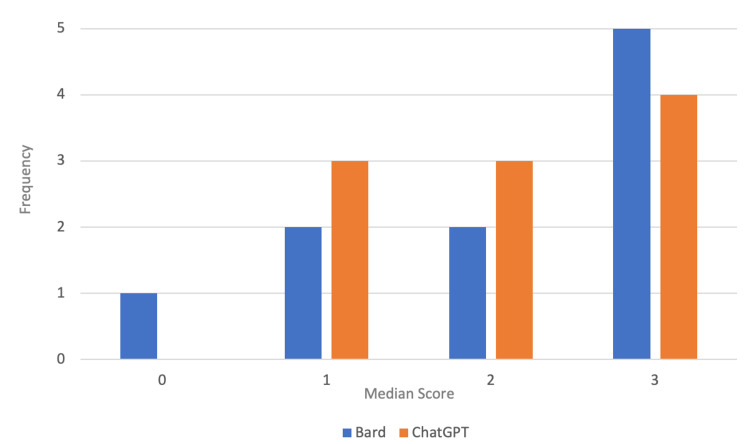
Frequency of median rater scores for each chatbot for the main questions

Figure 5 shows the frequency of median rater scores for each Chatbot across the questions. Five questions answered by Bard had a median rater score of 3, two had a median score of 2, two had a median score of 1, and one had a median rater score of 0. In contrast, four of the explanations provided by ChatGPT had a median rater score of 3, three had a median score of 2, three had a score of 1, and none had a score of 0. 

## Discussion

The potential for ChatGPT across medical education has been noted [5-7]. It has been suggested to facilitate evidence-based decision-making and generate differential diagnoses triggering self-directed learning on diagnoses unfamiliar to the student and as a knowledge resource [8] In the context of exam preparation, ChatGPT has been suggested to generate level-appropriate Objective Structured Clinical Examination (OSCE) scenarios (although notably marking domains and learning outcomes differ between institutions, and therefore it is hard to achieve reliability, validity, and calibration), even providing answers if prompted [15], and assist as a ‘virtual teaching assistant’ providing feedback and clarifying concepts [16].

This study aimed to assess the accuracy of responses and quality of explanations of Bard and ChatGPT in answering medical questions presented in the Resuscitation Council UK’s ILS paper focused on managing deteriorating patients and identifying causes and treating cardiac arrest. The performances of both Bard and ChatGPT were similar in answering the MCQs with similar scores achieved. Notably, despite having access to data across the web, neither of the LLMs answered all questions accurately. This suggests that there is still learning required of AI models in medical education. Evaluation of the explanations provided by the LLMs found that both Bard and ChatGPT were similar in the quality of explanation provided, with the majority of the explanations providing correct or useful information to aid learning of medical information. Further to this, it was noted that even in cases where the AI chatbots answered questions incorrectly, they were able to provide useful insights in their explanations.

Previous studies have sought to investigate the role of LLMs in other contexts of medical testing. A systematic review showed the impressive performance of generative AI models on standardised tests [17], including in the United States Medical Licensing Examination [18] and in specialised tests ranging from cardiology [19] to biochemistry [20] and general surgery [21]. However, there were cases where models failed exams such as the Taiwanese Family Medicine Board Exam [22]. In other studies, ChatGPT was shown to correctly answer two-thirds of all questions at a German state licensing exam level Progress Test [23] and performed at or near a passing level of 60% on a test of 350 items of the United States Medical Licensing Examination (USMLE) [18]. In other studies of medical physiology, ChatGPT secured around 75% [24,25]. In scoring each stem separately in our study, ChatGPT scored similarly at 75%. Although the difference was not noted to be significant, Bard scored 85% in our study.

The comparative performance of different chatbots in answering medical questions has been inconsistent in previous studies. ChatGPT outperformed Bard in a study of lung cancer questions [26] and neurosurgery board questions [27], which differed from our study. It is possible that performance may differ according to different subspecialties of medicine depending on the training algorithms the AI chatbots have been exposed to. Furthermore, a separate study by Kung et al. has shown that the performance of AI chatbots such as ChatGPT can vary depending on the exact prompt inputted for each question (e.g. forced justification) [18]. Thus, the choice of words for prompts could explain the differences in AI performance. Furthermore, the complexity of the input can further influence the performance accuracy of each of the LLMs in answering medical questions [9]. Improvement of AI models through training may allow them to be more accurate to further support learning.

The overall quality of the explanations provided by the LLMs for each question was found to be similar in our study. Interestingly, despite answering some questions incorrectly, the LLMs were able to provide useful and relevant information to aid in understanding the correct answers to these questions. This suggests that AI can support students in understanding material for questions even if answers are inaccurate; the challenge lies in students relying on AI to provide the correct answer without applying their own critical thinking as this may hinder understanding, particularly in cases where it may be used by students dishonestly to obtain answers. In fact, these AI LLMs may be more appropriately used to support the learning process and problem-solving without providing the answers, instead giving students information that guides them to deduce an answer for themselves. This supports constructivist theories that learning occurs as students form connections between new information to previous knowledge allowing them to build an understanding [28]. However, a further problem that students may face is that LLMs can often convincingly justify incorrect responses, in a phenomenon known as “hallucination” [17] limiting their utility for medical education. On the other hand, facing incorrect responses allows students to problem-solve and question the LLM responses, and through further discussion with educators students are able to develop their own knowledge. 

Our study supports the notion that LLMs can be increasingly utilised across the medical education curriculum, providing students with vast amounts of knowledge. However, in order to use LLMs appropriately and ensure students are still able to acquire the knowledge and understanding they need, students will benefit from engaging with educators who can guide students through complex concepts needed to answer medical questions and extend students’ learning beyond what they can achieve through independent use of LLMs. This is underpinned by Vygotsky’s social-cultural theory, which emphasises the importance of the cultural conditions under which knowledge is developed and places importance on the teacher-student relationship [29]; the purpose of tutor discussion is to capitalise on the fact that there is a difference between what a learner can learn independently and what they can learn with an experienced person and this difference is identified as Vygotsky’s Zone of Proximal Development [29,30]. Considering this in the context of our study, educators on Resuscitation Council courses would be able to support students' learning in addition to the use of LLMs.

Limitations

This study had notable limitations. This study was based on a small sample size of 40 true-false questions and 10 main questions from an ILS pre-course paper from the Resuscitation Council UK. These questions were selected since the Resuscitation Council UK’s life support courses are one of the only mandatory courses required for all UK medical doctors and thus assess the standard knowledge expected of all practicing physicians in the United Kingdom. However, the limited data set is arguably insufficient to comprehensively cover all aspects of resuscitation-based training. Also, whilst the questions are based on this topic they may only be representative of UK guidelines for management. Further studies with larger data sets are needed to comprehensively address this topic, including advanced life support and advanced cardiac life support guidelines. Our study focused only on ChatGPT-3.5 and Bard although other LLMs also exist including Claude-2 and Microsoft Bing. Furthermore, only the free version of ChatGPT - Chat GPT3.5 - was accessed for this study, and it is possible that ChatGPT-4 may perform differently in this setting, especially given its live internet browsing capabilities since mid-2023 [31]. A previous study of the Japanese licensing exam has shown that ChatGPT-4 outperformed ChatGPT-3.5 [32]. Additionally, although inter-rater reliability was noted to be strong, there may be subjectivity and human error in the evaluation of the explanations presenting a further limitation of our study.

## Conclusions

Our study provides insight into the suitability of AI LLMs to support students in learning concepts related to cardiopulmonary resuscitation. The results indicate a reasonable performance of ChatGPT and Bard in answering relevant questions but more importantly the ability of these LLMs to provide correct and insightful explanations that can aid learning. However, currently, these AI models require further training to improve their accuracy. The results of our study should be interpreted with caution given the limitations of the research design. As LLM technology evolves, future studies must be designed to address the limitations and provide further results that can be generalised in medical education.
